# The Smooth Away From Expected (SAFE) non-inferiority frontier: theory and implementation with an application to the D3 trial

**DOI:** 10.1186/s13063-023-07586-5

**Published:** 2023-08-25

**Authors:** Matteo Quartagno, Man Chan, Anna Turkova, Deborah Ford, Ian R. White

**Affiliations:** MRC Clinical Trials Unit, Institute for Clinical Trials and Methodology, University College London, 90 High Holborn, London, WC1V 6LJ UK

## Abstract

**Background:**

In a non-inferiority trial, the choice of margin depends on the expected control event risk. If the true risk differs from expected, power and interpretability of results can be affected. A non-inferiority frontier pre-specifies an appropriate non-inferiority margin for each value of control event risk. D3 is a non-inferiority trial comparing two treatment regimens in children living with HIV, designed assuming a control event risk of 12%, a non-inferiority margin of 10%, 80% power and a significance level (α) of 0.025. We consider approaches to choosing and implementing a frontier for this already funded trial, where changing the sample size substantially would be difficult.

**Methods:**

In D3, we fix the non-inferiority margin at 10%, 8% and 5% for control event risks of ≥9%, 5% and 1%, respectively. We propose four frontiers which fit these fixed points, including a Smooth Away From Expected (SAFE) frontier. Analysis approaches considered are as follows: using the pre-specified significance level (*α*=0.025); always using a reduced significance level (to achieve *α*≤0.025 across control event risks); reducing significance levels only when the control event risk differs significantly from expected (control event risk <9%); and using a likelihood ratio test. We compare power and type 1 error for SAFE with other frontiers.

**Results:**

Changing the significance level only when the control event risk is <9% achieves approximately nominal (<3%) type I error rate and maintains reasonable power for control event risks between 1 and 15%. The likelihood ratio test method performs similarly, but the results are more complex to present. Other analysis methods lead to either inflated type 1 error or badly reduced power. The SAFE frontier gives more interpretable results with low control event risks than other frontiers (i.e. it uses more reasonable non-inferiority margins). Other frontiers do not achieve power close (i.e. within 1%) to SAFE across the range of likely control event risks while controlling type I error.

**Conclusions:**

The SAFE non-inferiority frontier will be used in D3, and the non-inferiority margin and significance level will be modified if the control event risk is lower than expected. This ensures results will remain interpretable if design assumptions are incorrect, while achieving similar power. A similar approach could be considered for other non-inferiority trials where the control event risk is uncertain.

## Introduction

Non-inferiority (NI) clinical trials [1] have become the standard approach for investigating novel treatments that are unlikely to provide greater efficacy than standard of care, but may provide other benefits including better safety profiles, shorter regimens and lower costs [2].

A non-inferiority trial tests whether the new treatment’s efficacy is not unacceptably lower than the standard of care. Critical to this is the choice of the non-inferiority margin, which is the smallest non-acceptable loss of efficacy. The difference in the primary outcome between the two arms is estimated, together with an associated confidence interval, on a specific scale of interest. The choice of scale is key [3]. For example, for a binary outcome, one might specify the margin as an absolute risk difference, or a risk ratio. If the whole confidence interval for the treatment difference lies below the non-inferiority margin, then the novel treatment is considered non-inferior to the standard one [4].

Choosing the non-inferiority margin is a fundamental step in the design of a non-inferiority trial, and not a straightforward one [4, 5]. What people consider to be a non-acceptable difference may vary, depending on the specific settings (e.g. whether the outcome includes survival), but even for different people designing the same trial. Regulators have provided guidelines for choosing the non-inferiority margin [6, 7]. For both EMA and FDA, one key condition is that the non-inferiority margin needs to be chosen in order to guarantee the experimental treatment preserves some treatment effect against placebo; this is achieved by estimating M1, i.e. the effect of the active control against placebo, from available trials. The FDA also recommends a strategy based on defining M2, i.e. a certain proportion of the M1 effect that one should aim to preserve with the experimental treatment.

Whatever the strategy used to select the non-inferiority margin, its appropriateness is likely to depend on the assumed control event risk being close to the truth [8]. For example, for unfavourable outcomes, a smaller control event risk often means that a larger non-inferiority margin would not be tolerated. On the other hand, a larger control event risk leads to a loss of power if the non-inferiority margin is defined as a risk difference. Hence, choosing a single non-inferiority margin may result in difficulties in interpreting trial results or in loss of power if the control event risk turns out to be badly predicted.

Few strategies have been proposed to handle unexpected control event risks in non-inferiority trials [9–11]. One recently proposed approach is to define a non-inferiority frontier [8], i.e. a curve defining the most appropriate non-inferiority margin for each possible value of control event risk. Designing a trial using a non-inferiority frontier can make the trial resilient to unexpected event risks. In Quartagno et al. [8], we showed how to design a trial using a specific frontier, based on the power-stabilising transformation. However, this frontier can require a substantially (~20/40%) larger sample size compared to a standard trial designed with a fixed risk difference margin. The aim of this paper is therefore to present a less “expensive” frontier, that can protect against unexpected values of control risk at a lower cost in terms of sample size needed, or possibly without the need to increase the sample size. This may be of particular interest where funding is related to a pre-specified sample size. We develop our proposal for D3 (NCT04337450), a randomised non-inferiority clinical trial which aims to evaluate switching to a 2-drug therapy with dolutegravir (DTG)/lamivudine (3TC) given once daily in comparison with DTG-based triple-drug antiretroviral therapy (ART) in HIV-1 infected children and adolescents who are virologically suppressed on their current ART regimen.

The rest of this paper is organised as follows. We present the D3 trial and explain why methodology to handle unexpected event risks might be necessary. Then, in the “Methods” section, we present possible non-inferiority frontiers and describe approaches for implementation in D3. In the “Results” section, we show by means of analytical calculations which analysis method and frontier are preferable in terms of power and type 1 error, and, finally, we conclude with some discussion and a plan for future research.

## The D3 clinical trial

ART has hugely improved life expectancy for individuals living with HIV. Current HIV treatment guidelines recommend ART regimens consisting of three antiretroviral drugs: two nucleoside/nucleotide analogue reverse-transcriptase inhibitors (NRTIs) as a backbone, combined with an integrase strand transfer inhibitor (INSTI) as the “anchor” drug. DTG is currently recommended as a preferred anchor drug for treatment of children and adolescents.

Life-long ART is associated with challenges of treatment fatigue and long-term toxicities. Hence, research focus has shifted to investigating more tolerable and less toxic regimens that could improve the quality of life of patients, without compromising effectiveness.

The D3 trial aims to compare DTG/3TC dual therapy in HIV-1 infected children and adolescents who are virologically suppressed on their ART regimen to DTG-based three-drug ART as the recommended preferred first-line treatment for children [12]. At trial design, it was estimated that a total of 370 participants (185 per arm) would provide 80% power to exclude a fixed non-inferiority margin of 10 percentage points for the difference in the proportion of participants reaching the primary endpoint of confirmed viral rebound (2 consecutive HIV RNA ≥ 50 c/mL) by 96 weeks, assuming 12% risk in both arms, 10% loss to follow-up and a two-sided significance level of 0.05.

The expected control event risk and loss to follow-up assumptions were chosen in order to be conservative given the literature, in particular since larger control event risks imply larger sample sizes, a high control event risk was selected. The BREATHER trial recruited young people aged 8–24 years (including 61% aged ≥ 13 years) and all participants were on efavirenz-based ART; in the control arm (where 3-drug ART was given daily) the risk of virological rebound was 11% by 96 weeks [13, 14]. Given the lower age range in D3 and the use of dolutegravir as opposed to efavirenz, a failure rate of 12% was selected as the largest likely expected value of control event risk.

However, there remain potential problems with the design: if the control event risk is lower than expected, then a 10% non-inferiority margin might be considered too large to conclude the non-inferiority of the 2-drug arm. For example, FDA guidance on HIV switch trials [15] recommends a non-inferiority margin as small as 4 percentage points for an expected risk below 2%. We therefore investigated possible ways of changing the non-inferiority margin for lower-than-expected event risks by using a non-inferiority frontier, i.e. by defining the most appropriate non-inferiority margin corresponding to each value of control event risk.

## Methods

A non-inferiority frontier [8] is a curve that defines an appropriate value of the non-inferiority margin for different values of the true control event risk in a trial. For example, a trial that defines the non-inferiority margin as an absolute risk difference and plans to keep the margin fixed whatever the control event risk is using a fixed risk difference frontier. What we consider to be an appropriate non-inferiority margin for each control event risk does not vary compared to standard non-inferiority trials, i.e. it has to be based on clinical grounds, statistical considerations and regulatory guidelines. Here, we describe the features of the desired non-inferiority frontier and we list some possible alternatives for the D3 trial. In order to compare these frontiers in terms of power and type 1 error, we need to choose the best analysis method to implement them; thus, we select one of these frontiers (SAFE) and present alternative methods of analysing the trial according to such a frontier.

### Non-inferiority frontiers

While choosing the non-inferiority frontier, several considerations were made. First, as previously proposed, we considered the possibility to use the power-stabilising frontier, which is based on the arc-sine transformation [8]. However, this would have required a sample size 32% higher than originally planned, even if the expected control risk was correct, which was not considered feasible. Keeping the sample size fixed, power with such a frontier would have been as low as 70% at the expected control event risk. We therefore considered potential alternative frontiers and guided our choice with these considerations:

- Defining a frontier where the non-inferiority margin varies from the originally proposed 10 percentage point difference has some costs in terms of power; hence, we would define a frontier that only changes the non-inferiority margin within the range where the control event risk is reasonably likely to lie.
- We decided not to consider changing the non-inferiority margin for larger-than-expected event risks, as a control viral rebound risk (confirmed viral rebound >  = 50c/mL) larger than 12% was considered highly unlikely.
- We considered the originally selected non-inferiority margin of 10 percentage points reasonable for control event risk at or above 9%.
- In discussions with clinical colleagues, we chose to fix the non-inferiority margin at 5 percentage points for 1% control event risk and at 8 percentage points for 5% control event risk.

**Fig. 1:**
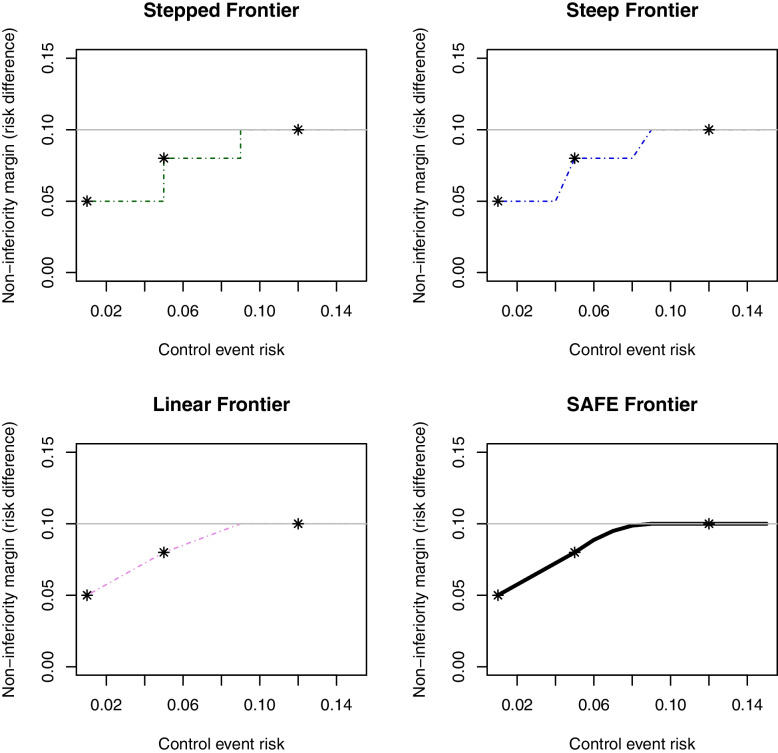
Non-inferiority frontiers considered for the D3 trial. The grey line indicates the 10% risk difference margin and the three stars are the points at which the non-inferiority margin was agreed in discussion with clinicians

These led us to consider the following frontiers, which are all plotted in Fig. 1.

#### Stepped frontier

This frontier is defined by a step function that assigns a non-inferiority margin of 8 percentage points when the control event risk is between 5 and 9% and 5 percentage points for control event risks at or below 5%. Its advantage is the straightforward definition and interpretation, while the disadvantage is the dichotomisation of the choice of non-inferiority margin for event risks larger or smaller than 5% or 9%.

#### Steep frontier

A similar, but less extreme, frontier allows for a non-immediate change in non-inferiority margin, but a continuous, though very steep, linear decrease in the non-inferiority margin from 10 to 8 percentage points between control event risks of 9 to 8% first, and from 8 to 5 percentage points between control event risks of 5 to 4%. The advantage of this frontier is that it avoids dichotomisation of the non-inferiority margin choice, though such a steep frontier might be considered unrealistic in terms of clinical significance.

#### Linear frontier

Another possibility is to allow for a linear decrease between control event risks of 9%, 5% and 1%, with two different slopes to achieve the desired non-inferiority margins of 8 and 5 percentage points. This is possibly more likely to reflect reasonable clinical opinion, but the function is still not differentiable at the points where the slope changes and initial explorations suggested that unsmooth frontiers could make type 1 error control difficult at the inflection points.

#### Smooth Away From Expected (SAFE) frontier

This frontier allows for a more gradual decrease in the non-inferiority margin for decreasing control risk. While the function remains linear between 5 and 1% event risks, the decrease from 9 to 5% is quadratic, to allow a gentler and smoother reduction of the non-inferiority margin for situations where the event risk is just below 9%.

We initially focus on the SAFE frontier (depicted as a black solid line in Fig. 1) and investigate possible ways to analyse a trial using this frontier. We later compare this to the other frontiers.

### Analysis methods for implementing the SAFE frontier

Here we list possible methods for implementing the SAFE frontier in the analysis of the D3 trial. While the first two methods were discussed in Quartagno et al. [8], the remaining two are novel proposals.

#### Post hoc modification of non-inferiority margin

One option is to simply select the non-inferiority margin at the analysis stage by looking at the SAFE frontier and selecting the non-inferiority margin corresponding to the observed control event risk. While this is a very straightforward and simple method, it is prone to inflation of type 1 error, as the same data are used twice: first, to select the non-inferiority margin and second, to test for non-inferiority using the same non-inferiority margin.

More generally, it would represent a post hoc adjustment, unlikely to be acceptable to regulatory agencies.

#### Reduce significance level

In order to control type 1 error, one option is to lower the significance level for testing. For example, instead of calculating 95% confidence intervals, one might calculate 97% or 99% intervals. The precise value could be chosen with the goal to achieve a type 1 error rate at or below the nominal value across the range of plausible control event risks covered by the SAFE frontier. In particular, for D3, simulations (which have been made available on GitHub, see below) suggested a 99% confidence interval would have to be computed.

While this method would control the type 1 error, it could be overly conservative as it involves changing the significance level across the whole frontier, including when the observed control event risk is the same as the expected control event risk.

#### Modify significance level if change non-inferiority margin

An alternative approach is to only modify the significance level if the non-inferiority margin has to be changed. For example, in D3 under the SAFE frontier, one would modify the significance level only if the control event risk is lower than 9% and hence the non-inferiority margin is modified.

#### Likelihood ratio test (LRT)

One final option is to perform a likelihood ratio test. The method works as follows:

- The unconstrained maximum likelihood estimates of the control and experimental arm risk are estimated (with the observed event risks), and the associated likelihood is calculated;
- The constrained maximum likelihood estimates are estimated, forcing the estimates to lie on the SAFE frontier, i.e. assuming inferiority, and finding the maxima with Newton-Raphson optimisation [16]. Once again, the associated likelihood is computed; and
- The test statistic is then calculated as minus twice the difference of the unconstrained and constrained log-likelihoods. Under the null hypothesis of the experimental arm being inferior to the control, this should follow a chi-square distribution.

Given this test, one can then build a test-based confidence interval, by providing the confidence interval for the risk difference that would correspond to the same p-value.

### Analytical calculation of operating characteristics

One way of comparing (i) analysis methods for SAFE and (ii) different frontiers would be to design a Monte Carlo simulation study, as is often done to evaluate novel statistical methods. However, due to both the relatively small sample size of D3 and the binary nature of both treatment and the outcome, in this specific example, we decided it was best to calculate the operating characteristics of various methods analytically, integrating over the probability of observing each number of events for any value of true control event risk. The advantage of this is that the results are exact and not affected by Monte Carlo error. Nevertheless, we present the plan for our analytical calculations here in a style similar to that recommended for Monte Carlo simulation studies [17].

#### Aim

To compare:

1. The operating characteristics of different methods of analysing trial results using the SAFE frontier and comparing them against those of a simple method keeping the non-inferiority margin fixed;
2. The operating characteristics of the best performing method in (1) when applied using various plausible non-inferiority frontiers including SAFE.

#### Data generating mechanisms

We consider all possible data arising from the D3 trial. In particular, we assume that 166 patients (the sample size before adjustment for expected attrition) are randomised to each of the two arms, and we estimate (i) the probability that precisely 0, 1, 2,…, 166 of them experience the outcome of interest in the control arm under the assumption that the true control event risk is between 1 and 15%; and (ii) the probability that precisely 0, 1, 2,…, 166 of the individuals in the experimental arm had viral rebound both under the null hypothesis implied by the non-inferiority frontier and under the alternative hypothesis that the true risk is the same as in the control arm.

#### Estimands

The risk difference for viral rebound is between the experimental and control arms.

#### Methods

For each of the possible combinations of events in the control and experimental arms, we apply the 4 methods to implement the SAFE frontier described in the “Analysis methods for implementing the SAFE frontier” section and also analyse keeping the non-inferiority margin fixed. For the best performing method, we repeat this using other non-inferiority frontiers described in the “Non-inferiority frontiers” section.

#### Performance measures

We focus on power and type 1 error for each true value of control event risk in the 1–15% range.

### Software

All the analyses performed here made use of the dani [18] R package, for the design and analysis of non-inferiority trials, which includes functions to help the design of trials using non-inferiority frontiers. The code used to produce the results is available on the GitHub page of the first author (https://github.com/Matteo21Q).

## Results

We first show the results of the analytical calculations presented in the previous section when comparing different analysis methods for the SAFE frontier. We then compare the results for three hypothetical datasets that could arise in the D3 trial, we discuss our preferred analysis method and we provide a table outlining its properties.

Finally, we provide the results of the analytical calculations comparing SAFE and the other frontiers when using the same analysis method.

### Comparison of analysis methods

**Fig. 2:**
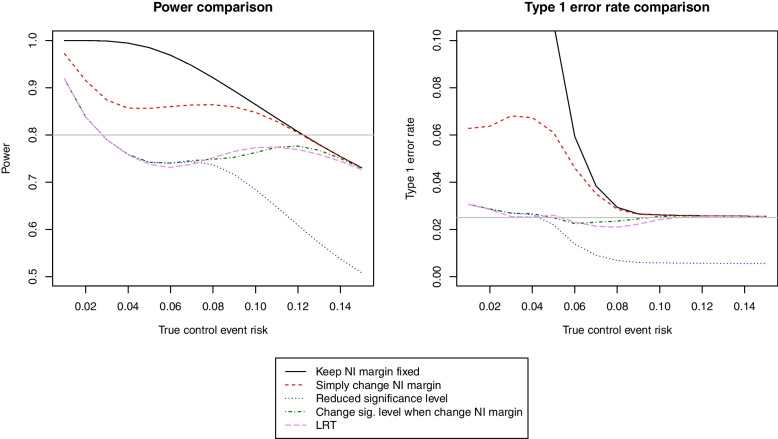
Comparison of various methods of implementing the SAFE frontier in terms of power and type 1 error. Note that for the fixed margin method, type 1 error is calculated against the null defined by the SAFE frontier

Figure 2 shows the results for power (nominal value 80%) and type 1 error rates (nominal value 2.5%).

#### Keep non-inferiority margin fixed

This is the most powerful strategy in situations where the control event risk was correctly predicted at the design stage of the trial (Fig. 2, left panel). However, in a situation where the true control event risk was lower than predicted, this strategy might lead to declaring non-inferiority when the upper bound of the confidence interval indicates that an absolute difference corresponding to a very large increase in relative risk might actually be plausible (see example 3 in Table 1 below). Hence, if we believe that the SAFE frontier truly defines our null hypothesis, the non-inferiority margin might not be appropriate for lower-than-expected event risks. If we were to evaluate type 1 error against the null hypothesis implied by the SAFE frontier, this method would badly inflate the type 1 error (Fig. 2, right panel).

**Table 1:** Results of three examples with various analysis methods, including the “keep NI margin fixed method” and four possible implementations of the SAFE frontier. The examples are (1) 22 events in both arms, (2) 15 events in the control and 22 in the experimental arm and (3) 2 events in the control and 10 in the experimental arm. The total sample size is fixed to 185 per arm in all examples, equal to the planned D3 sample size

	NI margin	Sig level (1-sided)	Confidence interval (2-sided 1-sig level*2)	*P*-value for NI test	Evidence for non-inferiority?
Example 1 (as expected, event risks 12% (C) and 12% (E)):					
Keep NI margin fixed	10%	2.5%	(−7.0%; +7.0%)	0.003	Yes
Post hoc modification of NI margin	10%	2.5%	(−7.0%; +7.0%)	0.003	Yes
Reduce significance level	10%	0.5%	(−9.2%; +9.2%)	0.003	Yes
Modify significance level if change NI margin	10%	2.5%	(−7.0%; +7.0%)	0.003	Yes
Likelihood ratio test		2.5%	(−7.9%; +7.9%)	0.006	Yes
Example 2 (lower control risk, event risks 8% (C) and 11% (E)):					
Keep NI margin fixed	10%	2.5%	(−3.4%; +9.4%)	0.017	Yes
Post hoc modification of NI margin	9.9%	2.5%	(−3.4%; +9.4%)	0.017	Yes
Reduce significance level	9.9%	0.5%	(−5.4%; +11.5%)	0.017	No
Modify significance level if change NI margin	9.9%	0.5%	(−5.4%; +11.5%)	0.017	No
Likelihood ratio test		2.5%	(−6.3%; +12.4%)	0.073	No
Example 3 (very low risk, event risks 1% (C) and 5% (E)):					
Keep NI margin fixed	10%	2.5%	(+0.4%; +8.0%)	0.002	Yes
Post hoc modification of NI margin	5.2%	2.5%	(+0.4%; +8.0%)	0.316	No
Reduce significance level	5.2%	0.5%	(−0.8%; +9.2%)	0.316	No
Modify significance level if change NI margin	5.2%	0.5%	(−0.8%; +9.2%)	0.316	No
Likelihood ratio test		2.5%	(+0.4%; +8.1%)	0.683	No

#### Post hoc modification of non-inferiority margin

Always changing the non-inferiority margin according to the frontier allows us to keep the power high for lower-than-expected control risks, but as expected it leads to substantial type 1 error inflation.

#### Reduce significance level

This strategy allows us to keep type 1 error under control, but this comes at huge costs in terms of power especially if the true control event risk is as expected. In order to reach the nominal 80% power level, one should increase the sample size by as much as 27%, which is close to the 32% increase needed for the power-stabilising frontier.

#### Modify significance level if change non-inferiority margin

This strategy allows us to recover a large amount of the power lost by always changing the significance level. The type 1 error rate, though, remains very close to the nominal 2.5% level.

#### Likelihood ratio test

Under this strategy, as expected given the underlying theory, type 1 error rate stays close to the nominal level whatever the true control event risk. Power is generally very similar to that of the previous strategy, so it remains quite high whatever the true control event risk.

#### Examples

If we assume 185 patients are randomised to the control arm and 185 patients to the experimental arm, and that there is 10% attrition, as hypothesised in the sample size calculation, we will observe the outcome for 166 patients in each arm. We consider three possible scenarios:

1. As expected (event risks 12% (C) and 12% (E)): 20 events are observed for both arms, i.e. participants meeting primary endpoint of confirmed viral rebound (2 consecutive HIV RNA ≥ 50 c/mL), for a 12.0% control and experimental arm event risk. This is exactly as assumed in the sample size calculations.
2. Slightly lower control risk (event risks 8% (C) and 11% (E)): 14 events (8.4%) are observed in the control arm, and 19 (11.4%) in the experimental arm.
3. Very low (event risks 1% (C) and 5% (E)): only 2 events (1.2%) are observed in the control arm, and 9 (5.4%) in the experimental arm.

Table 1 shows the results of the three examples using the various analysis methods. In example 1, when the observed event risks are exactly as expected, all methods produce similar results and lead to the same conclusions. However, in the second example, the analysis method matters: keeping the non-inferiority margin fixed leads to a borderline significant result, and using the post hoc modification of the non-inferiority margin method leads to the same conclusion, as the non-inferiority margin modification is minimal. The more conservative methods, though, lead to a *p*-value around 0.017 (“reduce significance level” and “modify significance level if change NI margin”, to be contrasted against a 0.005 significance level), or 0.073 (LRT with a 0.025 significance level).

Finally, example 3 is the one leading to the biggest differences between the method keeping the non-inferiority margin fixed and the other four, which are using the SAFE frontier. All methods using the SAFE frontier lead to similar results (large *p*-values, no evidence for non-inferiority), most likely because the control event risk is very extreme compared to expectations.

#### Comparison of methods

Among the strategies implementing the SAFE frontier, simply changing the non-inferiority margin and always reducing the significance level should be avoided due to serious issues with the type 1 error rate and power respectively.

The likelihood ratio method has optimal properties, but the method involving “changing the significance level when changing the non-inferiority margin” has comparable results in terms of both power and type 1 error rate and is much easier to explain and report. Therefore, for ease of interpretation, this is our favourite approach and the one we decided to take in D3.

Table 2 summarises information related to the application of “changing significance level when changing non-inferiority margin” method to the analysis of D3, including type 1 error rate, power and probability of changing the non-inferiority margin for all possible true values of control event risk. The non-inferiority margin and significance level to be selected based on each value of the observed control event risk are also summarised in the table. Interestingly, for the expected control event risk of 12%, only a minimal fraction of the nominal power was sacrificed to implement the SAFE frontier, so that power is still at approximately 79%. Note that this analysis method has now been pre-specified in the statistical analysis plan for D3.

**Table 2:** Choice of non-inferiority margin and significance level for the analysis of D3 based on observed confirmed viral rebound risk using the Smooth Away From Expected (SAFE) frontier and the “modify significance level if change non-inferiority margin” analysis method. The power, type 1 error and probability of changing the margin are shown for each true value of control event risk

**Control event risk (P0)**^**a**^	**1%**	**2%**	**3%**	**4%**	**5%**	**6%**	**7%**	**8%**	**9%**	**10%**	**11%**	**12%**	**13%**	**14%**	**15%**
**NI margin**	5.0%	5.8%	6.5%	7.3%	8.0%	8.9%	9.5%	9.9%	10.0%	10.0%	10.0%	**10.0%**	10.0%	10.0%	10.0%
**Significance level**	0.50%	0.50%	0.50%	0.50%	0.50%	0.50%	0.50%	0.50%	2.50%	2.50%	2.50%	**2.50%**	2.50%	2.50%	2.50%
**Power**	91.8%	83.8%	79.0%	75.9%	74.2%	74.1%	74.9%	75.9%	77.2%	78.6%	79.3%	**78.8%**	77.3%	75.2%	73.0%
**Type 1 error**	3.06%	2.86%	2.69%	2.72%	2.77%	2.65%	2.62%	2.52%	2.52%	2.58%	2.58%	**2.58%**	2.57%	2.56%	2.56%
***P* (change margin)**^**b**^	100%	100%	100%	99.9%	98.8%	95.0%	86.0%	71.4%	53.6%	36.2%	22.0%	**12.1%**	6.1%	2.8%	1.2%

The column in bold corresponds to the sample size calculation assumption made at the design stage

^a^The choice of non-inferiority margin and significance level will depend on the observed confirmed viral rebound risk. The power, type 1 error and probability of changing the margin depend on the true control event risk

^b^The probability of changing the margin is the probability that, for a given true control event risk, the observed control event risk will be lower than 9%, hence leading to using a non-inferiority margin in the analysis different from the originally planned 10%

### Comparison of non-inferiority frontiers

Here we compare the operating characteristics of different non-inferiority frontiers, analysed using the “Modify significance level if change non-inferiority margin” method. We planned to compare the stepped, steep, and linear frontiers to the SAFE frontier in terms of power and type 1 error for each possible true value of control event risk in the range considered.

**Fig. 3:**
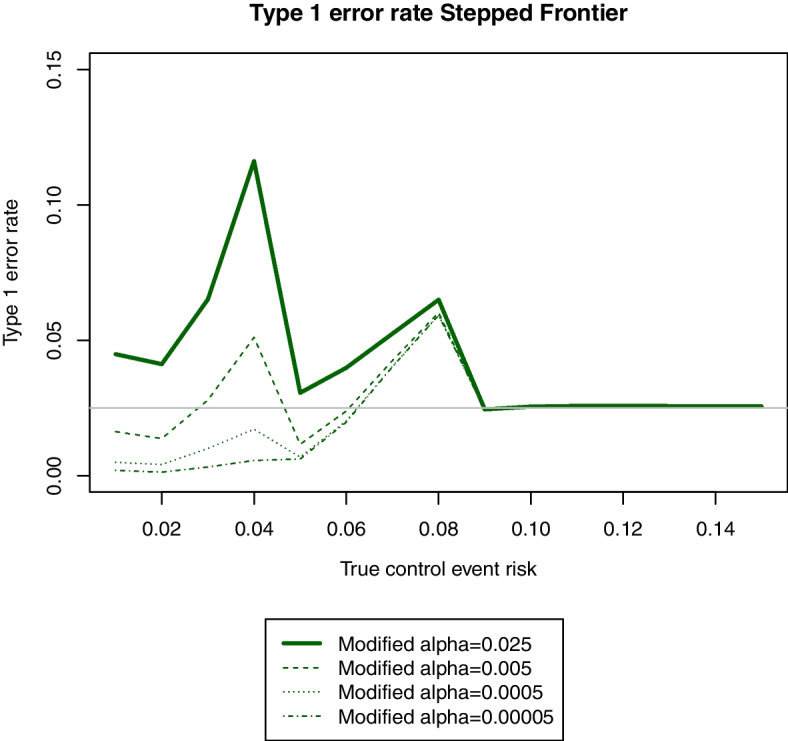
Type 1 error rate at different values of true control event risk when using the stepped frontier with the “modify significance level if change margin” method with different lower significance levels

When using the “Modify significance level if change non-inferiority margin” method, the first thing to do is to find the lower significance level that could control type 1 error for each frontier. While changing the significance level to 0.005 when changing the non-inferiority margin was enough for the SAFE and linear frontiers, it still did not control type 1 error for the stepped and steep frontiers. In fact, no reasonable significance level, i.e. no significance level that could maintain reasonable power, or no significance level higher than 0.00005, was enough to analyse the trial data controlling type 1 error using either the stepped or steep frontier. As an example, Fig. 3 shows the type 1 error curves for different values of modified significance level when using the stepped frontier. Therefore, it was only possible to use the linear and SAFE frontier when using this analysis method, suggesting that the smoothness of the frontier is a key element to be able to control type 1 error.

**Fig. 4:**
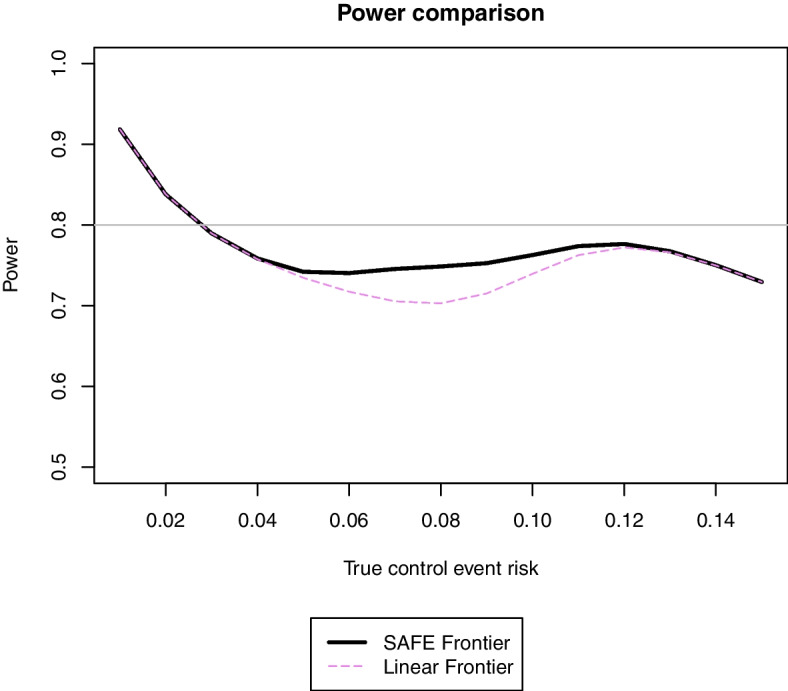
Comparison of power for analysing D3 using the linear or SAFE non-inferiority frontiers with different true control event risks

Figure 4 shows the results of the comparison between these two frontiers. Power is almost always lower for the linear frontier than for the SAFE frontier, mainly due to the stricter non-inferiority margins implied by the linear frontier for control event risks between 5 and 9%.

Note that the slight overinflation of type 1 error for very small event risks is not due to the use of frontiers, but rather to known limitations of Wald confidence intervals for very low event risks.

## Discussion

In this paper, we have proposed and compared different ways of implementing a non-inferiority frontier into the analysis of the D3 trial. We have seen how reducing the significance level in situations where the non-inferiority margin would be changed seems a good strategy both in terms of maintaining an approximately nominal type 1 error rate and keeping power at high levels, irrespective of the true control event risk.

We have additionally explored operating characteristics when using different frontiers and concluded that a Smooth Away From Expected (SAFE) frontier seems preferable, as a smoother transition to lower non-inferiority margins avoids kinks in the type 1 error function. This in turn leads to better power as well, when using an analysis method that lowers the significance level to control the type 1 error, like our preferred method. We only considered changing the frontier for lower-than-expected event risks, as D3 was designed conservatively and the risk of observing a higher control event risk seems low; hence, using the SAFE frontier can only make results less statistically significant than using a standard risk difference frontier, but it will make them more interpretable. However, a similar approach might be taken to protect against higher-than-expected risks. This would be aimed at preserving power rather than the interpretability of results.

When using non-inferiority frontiers, the usual recommendations and guidelines from regulators on how to select non-inferiority margins can still be followed by fixing the non-inferiority margin at a few selected points across the control event risk range, rather than using a single pre-specified margin selected on an expected event risk which may turn out to be wrong, either making results difficult to interpret or the trial underpowered.

In D3, when we decided to implement the SAFE, the sample size calculations had been carried out and more generally the trial had been already designed and was close to starting recruitment. This paper addressed such a scenario, where a frontier has to be found that can improve the interpretability of results without affecting power too much; however, ideally, non-inferiority frontiers should be taken into account in the sample size calculations. Future work will explore how to best do this, comparing various strategies. For example, one approach would involve increasing the sample size to achieve the nominal power level at the expected event risk, possibly using simulations, or analytical calculations similar to the ones performed here, to find the appropriate sample size. While this would not have changed the sample size much in D3, as power was very close to 80% when using the SAFE frontier, if we had considered increasing the sample size, a different frontier could have been selected, allowing for a potentially steeper reduction in the non-inferiority margin for decreasing control event risk. Aside from this, we do not expect the post hoc development of SAFE and implementation in D3 to raise the risk of bias of the trial, as these were developed before any trial data became available.

In D3, the confidence interval around the risk difference will be computed using bootstrap; however, because of the computational burden caused by bootstrap, in all the simulations here we used the simple Wald confidence interval. This is known to undercover for very low event risks and hence future work will investigate the impact of using alternative confidence interval computation methods, as the one proposed by Newcombe [19].

A methodology like the one presented here can be adjusted to be implemented in any non-inferiority trial with binary outcomes; however, arguably it becomes more and more appealing when the likely event risks lie in the very high (say > 80%) or very low (< 20%) range. This is because, in such situations, the difference between the smallest non-tolerable experimental arm event risk implied by a relative and absolute difference for an unexpected control event risk becomes larger, and hence it is more likely that keeping a fixed absolute difference margin could be considered problematic.

### Recommendations

1. Leaving the non-inferiority margin fixed whatever the control event risk should not be the default standard, because of its potential impact in terms of power loss and/or interpretability of results;
2. An alternative is to draw a SAFE non-inferiority frontier, with the goal to use certain target non-inferiority margins for certain levels of control event risk; these should be discussed with clinicians, considering how trade-offs differ for different values of control event risk. One possible approach is to:
  - Identify which values of control event risks are plausible;
  - Fix the desired value of non-inferiority margin at few values of control event risk (e.g. in D3 at 1% and 5% event risk); and
  - Draw a smooth frontier (as SAFE) continuously linking the various non-inferiority margins selected.
3. When implementing a SAFE frontier, the significance level should be reduced to a level that can control type 1 error at the nominal level; in order to maintain power, though, the significance level should only be modified when the non-inferiority margin is changed;
4. A method based on a likelihood ratio test has optimal properties as well, but the “modify significance level if change margin” method might be easier to interpret and communicate, and is therefore recommended; and
5. If possible, the frontier should be implemented into the design of the trial, so that the sample size calculation reflects it.

### Conclusions

Implementing the SAFE frontier in the analysis of non-inferiority trials can help improve efficiency and interpretability; in particular, its implementation in the D3 trial will lower the risk of testing for non-inferiority with an unacceptably large non-inferiority margin due to a lower-than-expected control event risk. Reducing the significance level when changing the non-inferiority margin can help preserve the nominal type 1 error rate without excessively affecting power.

## Data Availability

All the code used for simulations in this paper is available in the GitHub page of the first author: https://github.com/Matteo21Q.
